# Randomised, phase II trial comparing oral capecitabine (Xeloda®) with paclitaxel in patients with metastatic/advanced breast cancer pretreated with anthracyclines

**DOI:** 10.1038/sj.bjc.6600261

**Published:** 2002-05-06

**Authors:** D C Talbot, V Moiseyenko, S Van Belle, S M O'Reilly, E Alba Conejo, S Ackland, P Eisenberg, D Melnychuk, T Pienkowski, H-U Burger, S Laws, B Osterwalder

**Affiliations:** Cancer Research UK, Medical Oncology Unit, Churchill Hospital, Oxford OX3 7LJ, UK; Petrov Research Institute, St Petersburg, Russia; Department of Medical Oncology, University Hospital Ghent, Ghent, Belgium; Clatterbridge Centre for Oncology and RLUH Breast Unit, Merseyside CH63 4JY, UK; Department of Medical Oncology, University Hospital, 29010 Malaga, Spain; Department of Medical Oncology, Mater Misericordiae Hospital, Newcastle, NSW, Australia; Marin Oncology Associates, Inc., Suite 200, S. Eliseo Drive, Greenbrae, CA 94904, California, USA; Department of Oncology, Jewish General Hospital, 3755 Chemin de la côte-Ste-Catherine, Montreal H3T 1E2, Canada; Breast Cancer Clinic, Institute of Oncology, Warsaw, Poland; F Hoffmann-La Roche Ltd, Basel, Switzerland; Quintiles, Strasbourg, France

**Keywords:** anthracycline-resistant, breast cancer, capecitabine, fluoropyrimidine, paclitaxel

## Abstract

Capecitabine, an oral fluoropyrimidine carbamate, was designed to generate 5-fluorouracil preferentially at the tumour site. This randomised, phase II trial evaluated the efficacy and safety of capecitabine or paclitaxel in patients with anthracycline-pretreated metastatic breast cancer. Outpatients with locally advanced and/or metastatic breast cancer whose disease was unresponsive or resistant to anthracycline therapy were randomised to 3-week cycles of intermittent oral capecitabine (1255 mg m^−2^ twice daily, days 1–14, (22 patients)) or a reference arm of i.v. paclitaxel (175 mg m^−2^, (20 patients)). Two additional patients were initially randomised to continuous capecitabine 666 mg m^−2^ twice daily, but this arm was closed following selection of the intermittent schedule for further development. Overall response rate was 36% (95% CI 17–59%) with capecitabine (including three complete responses) and 26% (95% CI 9–51%) with paclitaxel (no complete responses). Median time to disease progression was similar in the two treatment groups (3.0 months with capecitabine, 3.1 months with paclitaxel), as was overall survival (7.6 and 9.4 months, respectively). Paclitaxel was associated with more alopecia, peripheral neuropathy, myalgia and neutropenia, whereas typical capecitabine-related adverse events were diarrhoea, vomiting and hand–foot syndrome. Twenty-three per cent of capecitabine-treated patients and 16% of paclitaxel-treated patients achieved a ⩾10% improvement in Karnofsky Performance Status. Oral capecitabine is active in anthracycline-pretreated advanced/metastatic breast cancer and has a favourable safety profile. Furthermore, capecitabine provides a convenient, patient-orientated therapy.

*British Journal of Cancer* (2002) **86**, 1367–1372. DOI: 10.1038/sj/bjc/6600261 www.bjcancer.com

© 2002 Cancer Research UK

## 

Every year more than 425 000 women in Europe and the USA are diagnosed with breast cancer (Black *et al*, 1997; Landis *et al*, 1999), nearly half of whom will develop metastatic disease (Lippman, 1998). The prognosis for women with metastatic and/or advanced disease is poor, with a median survival time of approximately 18 to 30 months from diagnosis (Perez, 1998). In these circumstances, the primary goal of therapy is palliation (Hortobagyi, 1998; Blum, 1999; Parfitt, 1999) and treatment usually involves hormonal therapy or chemotherapeutic agents (Hortobagyi, 1998; Marty *et al*, 1999).

The widespread use of anthracycline-containing regimens as adjuvant and first-line treatment for breast cancer has resulted in an increase in the number of patients presenting with disease that is resistant to anthracyclines (Marty *et al*, 1999). The use of M-phase inhibitors such as taxanes and vinorelbine in these patients is widely accepted. Response rates of 22 to 28% are reported for paclitaxel (Perez, 1998), 29 to 41% for docetaxel (Bonneterre *et al*, 1999; Trudeau, 1999) and 15 to 16% for vinorelbine in this setting (Degardin *et al*, 1994; Ibrahim *et al*, 1999). The duration of remission is approximately 4 to 5 months (Degardin *et al*, 1994; Ibrahim *et al*, 1999).

Capecitabine (Xeloda®) is a tumour-selective fluoropyrimidine carbamate designed to mimic continuous infusion 5-FU and to generate 5-FU preferentially in tumour tissue by exploiting the higher concentrations of thymidine phosphorylase (TP) found in malignant cells compared with normal cells (Miwa *et al*, 1998). Following oral administration, capecitabine passes intact through the intestinal mucosa and is rapidly and extensively metabolised via a sequential triple enzyme pathway. Capecitabine and its intermediate metabolites are not cytotoxic, and require conversion to 5-FU by TP. Moreover, since elevated TP concentrations correlate with a poor prognosis in breast cancer patients (Toi *et al*, 1997), capecitabine may be particularly effective in this group of patients (Frings, 1998).

The selective tumour activation of capecitabine has been confirmed in a trial of patients with colorectal cancer (Schüller *et al*, 2000). Patients were treated with capecitabine 1255 mg m^−2^ twice daily for 5 to 7 days before surgical resection of their primary tumour or metastatic lesions. Concentrations of 5-FU were found to be 3.2-fold higher in primary colorectal tumour tissue than in adjacent healthy tissue and 21 times higher than in plasma. Capecitabine has demonstrated considerable clinical activity in patients with heavily-pretreated, paclitaxel-resistant metastatic breast cancer, a patient population that previously had no established treatment options. In a phase II trial in this setting, capecitabine therapy resulted in a 20% response rate (with an additional 43% of patients achieving stable disease) and a median survival exceeding 1 year (Blum, 1999).

As an oral agent, capecitabine avoids the complications associated with continuous infusion 5-FU, including the need for central venous access and the medical complications this can introduce such as thrombosis, bleeding, infection and pneumothorax. In addition, oral agents are preferred by patients and may provide pharmaco-economic benefits (Liu *et al*, 1997; Borner *et al*, 2000).

The primary objective of the present study was to evaluate the overall response rate to capecitabine given either intermittently or continuously to female patients with advanced and/or metastatic breast cancer who either had failed or were resistant to treatment with an anthracycline-containing regimen. A standard, 3-h infusion of paclitaxel every 3 weeks, an approved and effective second-line therapy (Perez, 1998), was included in the study as a reference arm to minimise recruitment bias. Secondary objectives were to determine and evaluate additional efficacy parameters and the safety profile of each treatment arm.

## PATIENTS AND METHODS

### Eligibility

The study included female patients (⩾18 years old) with histologically or cytologically confirmed advanced and/or metastatic breast cancer. Initially, patients could have been treated with only one anthracycline-containing chemotherapy regimen in the adjuvant or metastatic setting. To increase recruitment, this was amended to include patients treated with ⩽2 regimens for metastatic disease, the last regimen containing an anthracycline. All patients were either anthracycline resistant (relapse within 6 months of completion of adjuvant therapy, initial response followed by disease progression while on the same therapy or disease progression on therapy without evidence of objective response) or anthracycline failing (stable disease after a minimum of four cycles, complete or partial response followed by disease progression within 12 months of treatment, or relapse 6 to 12 months after completion of anthracycline-based adjuvant treatment). Patients had at least one bidimensionally measurable lesion that had not been previously irradiated (new lesions in previously irradiated fields were accepted). Bone lesions, ascites and pleural effusions were not considered measurable. Patients were required to have a minimum indicator lesion size of ⩾20 mm for liver, soft tissue or other masses, or ⩾10 mm in lung, skin lesions or lymph nodes. Further inclusion criteria included a life expectancy of ⩾3 months and a Karnofsky Performance Status of ⩾70%.

Patients who had received prior treatment with taxanes or continuous/protracted administration of fluoropyrimidines were excluded. Patients with brain metastases were also excluded, as were patients who lacked physical integrity of the gastrointestinal tract or patients with malabsorption syndromes. Depressed bone marrow function (haemoglobin <9.0 g dl^−1^; granulocyte count <1.5×10^9^ l^−1^; platelet count <100×10^9^ l^−1^) and significant cardiac disease were excluded. Patients were not eligible for the study if screening evaluations revealed significant abnormalities in serum creatinine (⩾1.5×upper normal limit), serum bilirubin (⩾1.5×upper normal limit), alanine aminotransferase (ALT), aspartate aminotransferase (AST) or alkaline phosphatase (>2.5×upper normal limit or >5×upper normal limit in the case of liver metastases for all three parameters). Up to 10×upper limit of normal for alkaline phosphatase concentrations was permitted in patients with bone disease.

All patients provided written, informed, consent and the study was performed according to the Declaration of Helsinki and current amendments, and to the principles of Good Clinical Practice. The protocol and all amendments were approved by the appropriate research ethics committees.

### Study design and randomisation

The primary objective of this multicentre, open-label, randomised study was to evaluate the objective response rate to capecitabine therapy in patients with advanced and/or metastatic disease that had progressed following anthracycline treatment. Secondary objectives were to evaluate and compare the safety profiles of the three treatment arms and to determine and compare the efficacy profiles of the three treatment groups in terms of time to response, duration of response and time to disease progression.

Patients were randomised to three treatment arms in a 1 : 1 : 1 ratio. Patients were stratified according to anthracycline resistance or failure, as defined previously. Capecitabine was administered in 3-weekly cycles, continuously (666 mg m^−2^ twice daily without interruption) or intermittently (1255 mg m^−2^ twice daily, 2 weeks treatment followed by 1 weeks rest), for at least two cycles. Capecitabine was taken twice daily at 12±2-h intervals, within 30 min of breakfast and dinner, with water, rounded to the nearest dose that could be administered using 500 mg and 150 mg tablets. Paclitaxel 175 mg m^−2^ was administered as a 3-h i.v. infusion every 3 weeks, with appropriate premedication.

### Study assessments and analysis

Screening assessments were conducted within 2 weeks prior to the start of treatment and included medical history and general physical examinations, electrocardiogram and tumour assessment (CT scan, chest X-ray, bone scan and X-rays). Assessment of vital signs, physical measurements and general laboratory tests were conducted within 1 week prior to the start of treatment.

Tumour response was assessed based on World Health Organization (WHO) criteria (WHO Handbook, 1979) at screening, at weeks 6, 12 and 18, and at treatment discontinuation. In patients with objective tumour regression or stable disease who continued treatment beyond 18 weeks, subsequent tumour response was assessed at the discretion of the investigator. The primary endpoint was overall response rate (complete or partial response as best response). The secondary endpoints were defined as follows: time to disease progression (interval between first day of treatment and first recording of disease progression or death); time to response (interval between treatment start and the day a response (in confirmed responders) was first recorded); and duration of response in patients with complete or partial response (interval between treatment start and the first recording of disease progression).

Adverse events (graded as mild, moderate, severe or life threatening according to National Cancer Institute of Canada Common Toxicity Criteria, (NCIC CTC)) were recorded up to 28 days after the last dose of study drug. Hand–foot syndrome was graded 1–3 (Blum *et al*, 1999a).

### Dose modifications

Treatment interruption or dose reduction was not indicated for reactions unlikely to become serious or life threatening, for example alopecia. In patients receiving capecitabine, treatment was interrupted in the event of toxicities classified as grade 2 or higher. Dose modifications were applied after resolution to grade 1 or 0, as outlined in Table 1

**Table 1:**
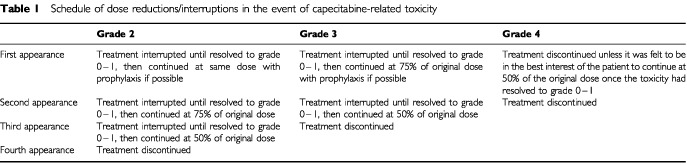
Schedule of dose reductions/interruptions in the event of capecitabine-related toxicity

.

In patients receiving paclitaxel who had a neutrophil count <1500 cells mm^−3^ and/or platelet count <100 000 mm^−3^ on the day scheduled for the next administration of treatment, therapy was delayed until recovery of neutrophil count ⩾1500 cells mm^−3^ and platelet count ⩾100 000 mm^−3^. The paclitaxel dose was reduced to 135 mg m^−2^ if patients experienced any of the following adverse events: febrile neutropenic episode, granulocyte count <500 cells mm^−3^ for ⩾15 days, platelet count <50 000 mm^–3^ associated with bleeding or platelet count <25 000 mm^−3^ with or without an episode of bleeding. If these toxicities recurred during a subsequent treatment cycle, the paclitaxel dose of 135 mg m^−2^ was further reduced by 25%. For all other toxicities dose modifications were the same as for capecitabine (Table 1). Patients with major hypersensitivity were withdrawn from study treatment.

### Statistical analysis

Descriptive analyses of efficacy and standard safety summaries were performed. Best overall responses were summarised by response rates and 95% Pearson–Clopper confidence intervals. Duration of response is analysed according to WHO criteria. It was planned to analyse time to disease progression or death, survival, duration of response and time to response according to Kaplan–Meier estimates, with survival and time to disease progression curves compared using the log-rank test. However, owing to the premature discontinuation of the trial, these analyses were not performed.

The planned sample size of 102 evaluable patients allowed for 34 patients to be randomised to each treatment arm. Following discontinuation of the continuous capecitabine arm (after inclusion of two patients in this arm), it was planned to close patient recruitment after enrolment of a total of 76 patients or 68 evaluable patients. Data were analysed and compared only for patients in the intermittent capecitabine and paclitaxel groups. The number of patients in the continuous capecitabine group (two) was too small for meaningful analysis.

## RESULTS

### Patient demography and disposition

The study was conducted from May 1996 to March 1997 at 18 centres in Australia, Belgium, Canada, France, Poland, Russia, Spain, the UK and the USA. During recruitment, data from a randomised, phase II study in colorectal cancer patients led to the selection of the intermittent capecitabine regimen for further clinical development (Van Cutsem *et al*, 2000), and therefore recruitment to continuous capecitabine treatment in the present study was stopped, creating a two-arm study. The recruitment target was 76 patients or 68 evaluable patients. This target was not reached because of clear preferences of the patients for either an oral investigational drug or an i.v. drug with established efficacy. Forty-four patients were randomised, 22 to intermittent capecitabine, 20 to paclitaxel and two to continuous capecitabine treatment. Owing to brain metastases diagnosed 1 day after randomisation, one patient randomised to paclitaxel never received treatment, and therefore this patient is not included in the treatment analysis.

Table 2

**Table 2:**
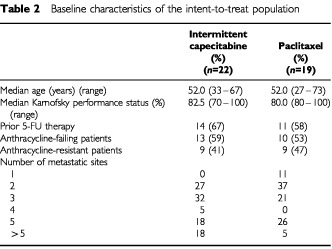
Baseline characteristics of the intent-to-treat population

summarises the baseline demographic and tumour characteristics, which were well balanced between treatment arms. All but two patients had ⩾2 (median two, range one to seven) metastatic sites. Almost all patients (86%) had received prior chemotherapy with cyclophosphamide, 61% had been treated with 5-FU-containing regimens and 68% had received anti-oestrogens.

Twenty-four patients discontinued treatment during the 18-week study period, with similar proportions discontinuing from the intermittent capecitabine (54%) and paclitaxel (63%) arms. The most frequent reason for treatment discontinuation was insufficient response/disease progression (66% of patients who discontinued therapy in both treatment arms). Ten of the 22 intermittent capecitabine patients and seven of the 19 paclitaxel patients completed the 18-week study period.

### Efficacy

The best response achieved during the treatment period was determined for the intent-to-treat population, and results are presented in Table 3

**Table 3:**
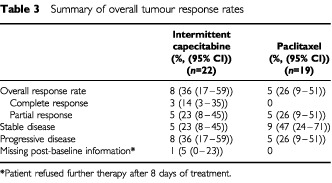
Summary of overall tumour response rates

. The primary endpoint, overall response rate (complete or partial response), was 36% (95% CI 17–59%) in the capecitabine group and 26% (95% CI 9–51%) in the paclitaxel group (not statistically different). Moreover, complete responses occurred in three patients treated with intermittent capecitabine but no patients in the paclitaxel group. In both treatment arms, responses generally occurred early in the course of treatment (week 6 or week 12 assessments), with the exception of one patient in the paclitaxel arm who first showed a response at week 18.

At the time of database closure, disease had progressed in three of the responding patients in each group. Disease progression was recorded at days 118, 187 and 288 in the capecitabine patients and days 178, 197 and 288 in the paclitaxel patients. Response duration was in excess of 18 weeks in five patients treated with capecitabine and two patients treated with paclitaxel. The median duration of response was in excess of 9.4 months in both treatment groups.

Time to disease progression was similar in the two treatment groups (median 3.0 months (95% CI 1.4–6.6) with capecitabine and 3.1 months (95% CI 2.5–6.5) with paclitaxel). Overall survival was similar in the two treatment groups (median 7.6 months, 95% CI 3.5–13.5 with capecitabine; 9.4 months, 95% CI 6.1–10.2 with paclitaxel).

### Safety

In both treatment arms almost all patients experienced treatment-related adverse events. Paclitaxel was typically associated with alopecia, paraesthesia, peripheral neuropathy and myalgia, whereas capecitabine was associated with more diarrhoea, vomiting and hand–foot syndrome. The incidence of nausea and stomatitis was similar in the two treatment arms (Table 4

**Table 4:**
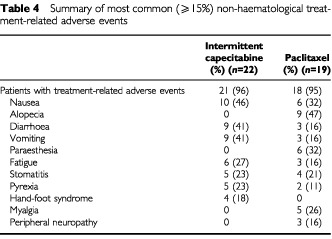
Summary of most common (⩾15%) non-haematological treatment-related adverse events

). The overall incidence of treatment-related grade 3 adverse events was 58% with paclitaxel and 23% with capecitabine. Grade 3 adverse events reported in the capecitabine group were: nausea (two cases), vomiting (two cases), hand–foot syndrome (two cases), constipation and neutropenia reported by the investigator as a clinical adverse event requiring medical intervention (one case each). In the paclitaxel arm, grade 3 adverse events were alopecia (five cases), and one each of paraesthesia, stomatitis, diarrhoea, vomiting, asthenia, pain, musculoskeletal pain and anaemia. There were four treatment-related grade 4 events in three patients: abdominal pain and diarrhoea (one capecitabine patient), aplasia reported by the investigator as a clinical adverse event (one paclitaxel patient) and neutropenia as a clinical adverse event (one paclitaxel patient).

A major difference between treatments concerned the incidence of grade 3/4 haematologic abnormalities, with substantially less myelosuppression reported in the capecitabine group (Table 5

**Table 5:**
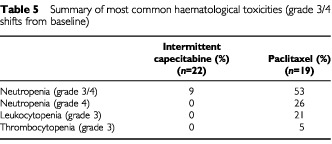
Summary of most common haematological toxicities (grade 3/4 shifts from baseline)

). The incidence of grade 3/4 shifts in neutropenia was markedly higher in the paclitaxel arm than in patients receiving capecitabine (53% *vs* 9%, respectively). There were no grade 3 or 4 shifts from baseline in alanine aminotransferase, aspartate aminotransferase, alkaline phosphatase, or bilirubin, except for one grade 3 shift in ALT in a patient receiving paclitaxel.

Five patients receiving intermittent capecitabine required treatment interruption and/or modification, but not discontinuation, to control the following adverse events: neutropenia (one patient), hand–foot syndrome (two patients), pyrexia (one patient) and hypotension plus pyrexia (one patient). No patients withdrew from capecitabine treatment because of adverse events. One paclitaxel patient required dose modification for neutropenia. In addition, treatment was discontinued in one patient receiving paclitaxel owing to treatment-related nausea and vomiting. There were no treatment-related deaths in either group. At database closure, 14 patients in the capecitabine arm and nine patients in the paclitaxel group had died.

Karnofsky Performance Status scores were generally stable or decreased moderately (10–20%) during the study. One paclitaxel patient had a clinically relevant decrease (⩽40%) in performance status. Improvement from baseline of ⩾20% was reported in three patients in the capecitabine group. A further two patients receiving capecitabine and three patients receiving paclitaxel showed ⩾10% increases.

## DISCUSSION

In this randomised, phase II study the continuous capecitabine treatment arm was abandoned following selection of the intermittent capecitabine regimen for further development. This selection was based on the results of a randomised, phase II trial in 109 colorectal cancer patients, in which intermittent capecitabine resulted in a more favourable time to disease progression than the other regimens tested. The intermittent schedule also exhibited an acceptable toxicity profile over a wider dose range than the continuous regimen, enabled exposure to a higher dose intensity and included a 1-week drug-free period, which was considered more appealing to patients. In addition to the discontinuation of one treatment arm, the present study did not reach the target patient number owing to recruitment issues. Candidate patients expressed a strong preference for either the investigational oral agent or the established i.v. treatment and therefore refused to be randomised.

Results of the study indicate that capecitabine, administered as an intermittent regimen, shows similar efficacy to paclitaxel in patients with metastatic and/or advanced breast cancer previously exposed to anthracyclines. The tumour response rate was 36% in patients treated with intermittent capecitabine and 26% in patients treated with paclitaxel, although the confidence intervals overlapped, owing to the small sample sizes. Moreover, three patients treated with capecitabine, but no patients in the paclitaxel arm, achieved complete responses. Responses tended to occur earlier in patients receiving capecitabine. The other secondary variables indicated similar efficacy in the two treatments. The validity of this comparison is supported by the response rate for paclitaxel patients, which was within the 22 to 28% range reported in the literature (Perez, 1998).

Almost two-thirds of patients (61%) had received prior treatment with bolus 5-FU, indicating that capecitabine may be useful in the treatment of patients whose disease has progressed during or following 5-FU therapy. This observation is consistent with results of preclinical studies, which indicated that capecitabine has anti-tumour activity even in cell lines that are resistant to 5-FU (Cao *et al*, 1997). There is some evidence of a correlation between high TP activity and resistance to 5-FU (Danenberg *et al*, 1997). Therefore, a consequence of the unique, TP-mediated mechanism of action of capecitabine may be incomplete cross-resistance to conventional 5-FU in tumours overexpressing TP.

The high activity of intermittent capecitabine in pretreated breast cancer has been observed in two other phase II trials in metastatic/advanced breast cancer patients who had failed or were resistant to taxane therapy. In the first trial, which included 162 patients (Blum *et al*, 1999a), the overall response rate was 20% (with a further 43% achieving stable disease) and the median survival was 12.6 months. Additionally, 47% of patients with significant pain at baseline showed a durable reduction in pain scores of at least 50%. In a subgroup of 42 patients resistant to both paclitaxel and doxorubicin, the response rate was 29%. In the second study (Blum *et al*, 1999b), which included 74 patients pretreated with either paclitaxel or docetaxel, the response rate was 25% (27% in patients pretreated with paclitaxel and 20% in patients pretreated with docetaxel). The activity of capecitabine as second- or third-line therapy thus compares favourably with trials of some other agents reported in the literature. In a study of vinorelbine in patients pretreated with paclitaxel, there were no responses (Fazeny *et al*, 1996). Intermittent capecitabine therapy has also been evaluated as first-line treatment for breast cancer in a randomised, phase II study in 95 women aged ⩾55 years (O'Shaughnessy *et al*, 1998; Aapro, 2000). The results indicated that capecitabine achieved a response rate of 30%. Patients receiving the reference arm i.v. regimen of CMF achieved a response rate of 16% (not significant). The cumulative weight of this evidence indicates that capecitabine is an effective agent for the treatment of breast cancer.

In the present study, capecitabine appeared to have safety benefits compared with paclitaxel. The majority of adverse events were graded as mild or moderate in intensity. The most common side effects of capecitabine therapy were diarrhoea, nausea, vomiting, fatigue and hand–foot syndrome. Grade 3 adverse events were reported in 23% of capecitabine patients, but only one patient experienced a grade 4 adverse event. Of note, no significant alopecia was reported in patients receiving capecitabine. Myelosuppression was rare, with no cases of grade 2, 3 or 4 thrombocytopenia. Adverse events were controllable by treatment interruption and, where necessary, dose reduction, along with patient education and counselling. Furthermore, it has been shown that efficacy is not compromised if the dose is reduced from the standard dose for adverse events (Hoff, 2000; O'Shaughnessy and Blum, 2000). None of the patients was prematurely withdrawn from the study due to treatment-related adverse events, and there were no treatment-related deaths. The incidence of laboratory abnormalities was very low, despite the high incidence of bone and liver lesions. This safety profile is very similar to that seen in other clinical studies investigating capecitabine (O'Shaughnessy *et al*, 1998; Blum, 1999; Blum *et al*, 1999a,b; Aapro, 2000; Van Cutsem *et al*, 2000).

The safety profile observed with paclitaxel, which was consistent with the previous reports (Gelmon, 1994; Gelmon *et al*, 1999) contrasted with that of capecitabine. Alopecia, peripheral neuropathy and paraesthesia were common with paclitaxel. Alopecia was reported in half of the paclitaxel group, with half of the cases classified as grade 3. Myelosuppression, which is known to be the dose-limiting side effect of this compound (Gelmon, 1994), was common, with 53% of patients experiencing grade 3/4 neutropenia.

The convenient, oral administration of capecitabine combined with its efficacy and manageable toxicity profile make it an attractive agent for outpatient use. Myelosuppression was rare, making capecitabine a suitable agent for pretreated patients. Capecitabine offers a convenient, patient-orientated therapy and enables patients to control their treatment. Results of this study indicate that capecitabine achieves efficacy in a similar range to that of paclitaxel, and is well tolerated as treatment for metastatic breast cancer failing anthracycline therapy, while offering the advantages of oral administration. Capecitabine is also being evaluated in combination with paclitaxel and docetaxel with the aim of increasing efficacy in patients with metastatic breast cancer.
